# Effect of AcHERV-GmCSF as an Influenza Virus Vaccine Adjuvant

**DOI:** 10.1371/journal.pone.0129761

**Published:** 2015-06-19

**Authors:** Hyo Jung Choi, Yong-Dae Gwon, Yuyeon Jang, Yeondong Cho, Yoon-Ki Heo, Hee-Jung Lee, Kang Chang Kim, Jiwon Choi, Joong Bok Lee, Young Bong Kim

**Affiliations:** 1 Department of Bio-industrial Technologies, Konkuk University, Neungdong-ro, Gwangjin-gu, Seoul, Republic of Korea; 2 College of Veterinary Medicine, Konkuk University, Neungdong-ro, Gwangjin-gu, Seoul, Republic of Korea; The University of Adelaide, AUSTRALIA

## Abstract

**Introduction:**

The first identification of swine-originated influenza A/CA/04/2009 (pH1N1) as the cause of an outbreak of human influenza accelerated efforts to develop vaccines to prevent and control influenza viruses. The current norm in many countries is to prepare influenza vaccines using cell-based or egg-based killed vaccines, but it is difficult to elicit a sufficient immune response using this approach. To improve immune responses, researchers have examined the use of cytokines as vaccine adjuvants, and extensively investigated their functions as chemoattractants of immune cells and boosters of vaccine-mediated protection. Here, we evaluated the effect of Granulocyte-macrophage Colony-Stimulating Factor (GmCSF) as an influenza vaccine adjuvant in BALB/c mice.

**Method and Results:**

Female BALB/c mice were immunized with killed vaccine together with a murine GmCSF gene delivered by human endogenous retrovirus (HERV) envelope coated baculovirus (1×10^7^ FFU AcHERV-GmCSF, i.m.) and were compared with mice immunized with the killed vaccine alone. On day 14, immunized mice were challenged with 10 median lethal dose of mouse adapted pH1N1 virus. The vaccination together with GmCSF treatment exerted a strong adjuvant effect on humoral and cellular immune responses. In addition, the vaccinated mice together with GmCSF were fully protected against infection by the lethal influenza pH1N1 virus.

**Conclusion:**

Thus, these results indicate that AcHERV-GmCSF is an effective molecular adjuvant that augments immune responses against influenza virus.

## Introduction

The World Health Organization declared in 2009 that infections caused by a new strain of influenza virus—H1N1—had reached pandemic proportions, an assertion confirmed by laboratories in more than 214 countries and overseas territories [1]. The world is now in the post-pandemic period, and the H1N1 (2009) virus is expected to continue to circulate as a seasonal virus for years to come, accompanied by substantial additional morbidity, mortality, and economic losses [2].

Vaccination is clearly the most effective means for preventing and controlling influenza viral infection [3]. Nearly all commercial vaccines against influenza virus worldwide today are produced in eggs or cultured mammalian cells [4, 5]. The use of these platforms for the production of influenza vaccine, however, is associated with several potential problems, including the vulnerability of the material supply, the necessity for a selection of strains, and the iterative, often time-consuming production process [6]. One strategy for overcoming these obstacles is to produce virus-like particles using key viral structural proteins, such as hemagglutinin (HA), neuraminidase (NA), nucleoprotein (NP), and membrane protein (M) [7–9]; another is to develop an appropriate vaccine adjuvant.

Granulocyte-macrophage Colony Stimulating Factor (GmCSF), a member of the cytokines, is known to play a role in augmenting the immune response, particularly the function of professional antigen-presenting cells such as dendritic cells and macrophages, making GmCSF useful as a vaccine adjuvant [10–16].

Recent accumulating evidence supports the idea that baculoviruses carrying mammalian cell promoters can mediate expression of foreign genes in a variety of primary, established mammalian cells and animal models [17, 18]. Owing to their highly efficient gene-delivery mechanism, baculoviruses have drawn considerable interest as novel vectors for target gene delivery [19]. We previously reported that delivery of antigen-encoding DNA using a non-replicable baculovirus vector as a nano-carrier improved the efficacy of vaccines and shown that incorporating the envelope glycoprotein of human endogenous retrovirus (HERV-W) in recombinant baculovirus improves exogenous gene delivery into human cells [20–22].

BALB/c mice, these animals fits evaluation of immune response after baculovirus immunization and has suitable sensibility for mouse adapted pH1N1 virus [22–25]. For these purposes, we selected BALB/c mice to enhance the immunogenicity and reduce the antigen dose or immunization frequency required for protective immunity, we constructed a baculovirus vector carrying *Mus musculus* GmCSF (AcHERV-GmCSF) and tested its molecular adjuvant efficacy with killed—pH1N1 influenza—vaccine.

## Materials and Methods

### 1. Ethics statement

Animal husbandry and experimental procedures confirmed by the Konkuk University Institutional Animal Care and Use Committee (IACUC approval No.: KU14082) and performed in strict accordance with the Guide for the Care and Use of Laboratory Animals of the National Institutes of Health [26].

### 2. Cells

Sf9 (Invitrogen, CA, USA) cells were maintained at 27°C in Sf-900 medium (Invitrogen, CA, USA) supplemented with 1% antibiotics/antimycotics (Gibco-BRL, CA, USA). 293TT cells (kindly donated by Dr. Schiller, National Cancer Institute, NIH, USA) were cultured in Dulbecco’s modified Eagle’s medium (DMEM; Gibco-BRL) supplemented with 10% fetal bovine serum (FBS; Gibco-BRL) and 400 μg/mL hygromycin B (Invitrogen) [27].

Madin-Darby canine kidney (MDCK; American Type Culture Collection, Manassas, VA, USA) cells were grown in Eagle’s minimum essential medium (MEM; Gibco-BRL) containing 10% FBS and 1% penicillin and streptomycin. The cells were maintained in a humidified 5% CO_2_ atmosphere at 37°C.

### 3. Mice and viruses

Female BALB/c mice (18.5±0.9 g), aged 8 weeks (n = 110, n refers to number of animals, mouse VAF report indicated that the mice were free of known viral, bacterial and parasitic pathogens) were purchased from Orient-Bio (Gyeonggi-do, Korea) and housed under filter top conditions with water and food (All mice were allowed access to water and food supplied freely) supplied *ad libitum* with an inverse 12 hours day-night cycle with lights on at 8:30pm in a temperature (22±1°C) and humidity (55±5%) controlled room. All cages contained wood shavings and bedding.

Mouse-adapted influenza virus type A/CA/04/2009 (ma-pH1N1) was kindly provided by the International Vaccine Institute (IVI, Seoul, Korea). The virus was maintained in 10-day-old embryonated eggs. After incubating for 3 days and chilling at 4°C for 12 hours, the allantoic fluid was harvested, aliquoted, and stored at -80°C until use.

### 4. Construction of recombinant baculoviruses expressing GmCSF

A recombinant baculoviral vector expressing HERV *env* (pFastBac1-HERV) was previously constructed by inserting a synthetic, codon-optimized envelope gene of HERV type W (GenBank accession number NM014590; GenScript, Piscataway, NJ, USA) into pFastBac1 (Invitrogen) [21].

The *M*. *musculus* GmCSF (GmCSF) gene (GenBank accession number X03019.1) in pcDNA3.1 vector (pcDNA3.1-GmCSF), kindly provided by NBM (Iksan, Korea), was subcloned into HERV-expressing pFastBac1 under the control of the hEF1α (human elongation factor-α) promoter (pFBHERV-GmCSF).

Recombinant baculoviruses were produced using the Bac-to-Bac baculovirus expression system (Invitrogen) according to a manufacturer’s instructions. The recombinant baculovirus, AcHERV-GmCSF was further amplified by propagation in Sf9 cells. The supernatant from the cells were loaded on top of 30% sucrose, and purified by ultracentrifugation at 40,000 rpm at 4°C for 1 hour in a SW50.1 rotor (Beckman Coulter Inc., Brea, CA, USA). The virus pellet was suspended in phosphate-buffered saline (PBS) and used for immunization.

### 5. Expression of GmCSF in mammalian cells

For mRNA quantification, 293TT cells were infected with AcHERV-GmCSF at a multiplicity of infection (MOI) of 10, and centrifuged to separate supernatants and lysates 48 hours after infection. Total RNA was isolated from the lysates using RNeasy mini kit (Qiagen, Valencia, CA, USA) and treated with DNase I (Promega, Fitchburg, WI, USA). cDNA was synthesized from total RNA using M-MuLV reverse transcriptase (Bioneer, Daejeon, Korea). GmCSF mRNA expression levels were determined by reverse transcription-polymerase chain reaction (RT-PCR) using the primer pair 5’-tga cat gcc tgt cac gtt gaa t-3’ (sense) and 5’-ggt agt agc tgg ctg tca tgt-3’, generating a 164-bp product. 18s ribosomal RNA (rRNA), used as an endogenous control, was amplified using the primer pair 5’-gtt ccg acc tat aac gat gcc-3’ (sense) and 5’-tgg tgg tgc cct tcc gtc aat-3’ (antisense).

Baculovirus infected 293TT cells were harvested together with the media and centrifuged to separate lysates and supernatant. The expression of GmCSF was determined in the lysates and supernatants using a mouse GmCSF ELISA Set (BD Biosciences, San Jose, CA, USA), according to the manufacturer’s instructions.

For immunofluorescence analyses, monolayers of 293TT cells seeded on glass slides in a 4-well plate were infected with AcHERV-GmCSF at an MOI of 10. Seventy-two hours after transduction, cells were fixed by incubating in a 4% formaldehyde/PBS solution for 20 minutes. After washing three times with PBS, cells were incubated with a mouse monoclonal anti-GmCSF antibody (1:200; BD Biosciences) for 2 hours at 37°C followed by incubation with fluorescein isothiocyanate (FITC)-conjugated goat anti-mouse IgG secondary antibody (1:200; Santa Cruz Biotechnology, Santa Cruz, CA, USA). Images of immunostained cells were acquired using an inverted microscope (Eclipse Ti-U; Nikon, Japan).

### 6. Animal experiments

These experiments were performed in strict accordance with the Guide for the Care and Use of Laboratory Animals of the National Institutes of Health [26] and carried out by following designed experimental timelines (S1 Fig). All surgery was performed on sterilized dissecting pan under mice mixture of tiletamine and xylazine anesthesia (50 and 5 mg/kg of body weight, respectively), and all efforts were made to minimize suffering.

#### Hematological analysis

GmCSF function was tested by performing hematological analyses of BALB/c mice (n = 2/group) immunized with 1×10^7^ focus-forming units (FFU) of AcHERV-GmCSF or 1×10^7^ FFU of wild-type Autographa californica multicapsid nucleopolyhedrovirus (AcMNPV) baculovirus, or injected with 100 μl of PBS. Blood samples were collected at 5-day intervals from the jugular vein of individual mice into tubes containing K2 EDTA (BD Microtainer, Franklin Lakes, NJ, USA). Total white blood cells and red blood cells were counted using a hematology analyzer (FORCYTE; Oxford Science, Oxford, CT, USA). The proportion of neutrophils, lymphocytes, monocytes, and eosinophils among total white blood cells was used as a hematological index.

#### Determination of effective dose of killed whole virus vaccine

The effective dosage of killed whole-virus vaccine was determined by ELISA and hemagglutination inhibition (HAI) assay. In brief, 8-week-old BALB/c mice (n = 3/group) were immunized by intramuscular injection of serially diluted (1.0–0.1 μg), killed vaccine together with 1×10^7^ FFU AcHERV-GmCSF; as a control, mice were immunized with 2 μg of killed vaccine or 1×10^7^ FFU AcHERV-GmCSF only at the same time points. On days 7, blood was collected from the jugular vein, centrifuged at 1500 rpm for 30 minutes, and the supernatant was transferred to a new microfuge tube for ELISA and hemagglutination inhibition (HAI) assay.

#### Evaluation of GmCSF adjuvant effect

Eight-week-old BALB/c mice (Female, n = 16/group) were divided into five immunization groups: (1) PBS control (100 μl), (2) AcHERV-GmCSF only (1×10^7^ FFU), (3) low-dose vaccine only (0.2 μg killed vaccine), (4) high-dose vaccine only (2.0 μg killed vaccine), and (5) vaccine plus AcHERV-GmCSF adjuvant (0.2 μg killed vaccine together with 1×10^7^ FFU AcHERV-GmCSF) and were given i.m. injection. On days 7, blood was collected from the jugular vein, centrifuged at 1500 rpm for 30 minutes, and the supernatant was transferred to a new microfuge tube.

Two weeks after immunization, mice (n = 13/group) were transferred to a biological safety level 2 facility, where they were sedated and challenged intranasally with mouse-adapted influenza virus A/CA/04/2009 (ma-pH1N1) at a 10×LD_50_ dose. One day after virus challenge, 4 mice from each group were separated for lung titer measurement and histological analysis. The mice in PBS group were sacrificed on 6 dpi and the rest of groups were sacrificed on 7 dpi to collect lung tissue. The remaining 9 mice per group were monitored for weight loss and survival rate for 12 consecutive day.

All surgery was performed on sterilized dissecting pan under mixture of tiletamine and xylazine anesthesia (50 and 5 mg/kg of body weight, respectively) in the light phase, and all efforts were made to minimize suffering.

A 10×LD_50_ dose challenge typically results in severe disease characterized by huddling, ruffled fur, lethargy, anorexia leading to weight loss, and death. Therefore, mice were monitored for weight loss and survival (twice per day) for 12 consecutive days. In case of mice showed both typical infection symptoms and weight loss over 25%, were humanely euthanized using carbon dioxide under condition of mixture of tiletamine and xylazine anesthesia (50 and 5 mg/kg of body weight, respectively) according to the NC3Rs ARRIVE guidelines for the euthanasia of animals.

### 7. Immunological assays

Each well of a 96-well plate was coated by incubation with 8 HAUs (hemagglutination units) of inactivated, diluted influenza virus pH1N1 (512 HAU/50 μl) for 16 hours at 4°C. After washing with PBS, serially diluted mouse sera (60 μl/well) was added to each well and were incubated for 2 hours at room temperature followed by subsequent washing. Horseradish peroxidase (HRP)-conjugated goat anti-mouse IgG antibody (1:2000; Santa Cruz Biotechnology) was then applied and incubated for 1 hour at room temperature. After subsequent washing, TMB (3,3’,5,5’-tetramethyl benzidine) substrate solution (Bio-Rad, Hercules, CA, USA) was added followed by application of 1N H_2_SO_4_ to stop the reaction. Color development was measured spectrophotometrically at 450 nm. Results are expressed as reciprocals of the final detectable dilution.

Anti-HA inhibition titers in HAI assays were measured by incubation with 4 HAIs of virus with 2-fold diluting heat-inactivated sera in V-bottom 96-well plates and incubating with 4 HAIs of virus for 40 minutes at room temperature, followed by incubation with 1% chicken red blood cells for 40 minutes at room temperature. The HAI titer is presented as the reciprocal of the highest dilution of serum that completely inhibited hemagglutination.

The production of interferon gamma (IFN-γ) from the splenocytes of immunized mice was detected by ELISPOT assay kit (BD Biosciences), as described by the manufacturer. Briefly, One day before splenectomy, a 96-well membrane plate was coated with 0.2 μg of mouse IFN-γ capture antibody and blocked with 10% FBS at 37°C. Randomized mice (n = 3 per group) were sacrificed under mixture of tiletamine and xylazine anesthesia (50 and 5 mg/kg of body weight, respectively) and splenectomy was performed with efforts to minimize suffering. Enucleated spleens were grinded on 40 μm nylon cell strainer (BD Falcon) and splenocytes were treated with RBC lysis buffer (Sigma-Aldrich). Splenocytes (1×10^6^ cells/well) in 100 μl of RPMI-1640 medium were applied in each well, stimulated with inactivated influenza virus pH1N1, and incubated for an additional 24 hours at 37°C. Plates were then washed with PBS containing 0.05% Tween-20 and treated with 20 ng of biotinylated mouse IFN-γ detection antibody for 2 hours. Streptavidin-alkaline phosphatase was then added to the wells, and color was developed with an AEC substrate reagent (BD Biosciences). The number of spots was counted using an ELISPOT reader (AID ElispotReader ver.4; AID GmbH, Straßberg, Germany).

### 8. Titration of virus in the lungs of challenged mice *in vitro*

Six days or seven days after challenge, separated mice (n = 4/group) were sacrificed and their lung tissue was collected in 3 ml PBS containing 2% gentamycin. Collected lungs were homogenized for approximately 1 minute using a hand-held tissue homogenizer (Biospec Products, Bartlesville, OK, USA), and centrifuged to remove debris. The resulting supernatant was mixed with 10-fold diluted MDCK cell monolayers in 96-well tissue culture plates and incubated for 2 days at 37°C. The virus titer was calculated using the Reed-Muench formula and was expressed as log10 TCID_50_ (median tissue culture infective dose) per milliliter.

### 9. Statistical analysis

All statistical analyses were performed using SigmaPlot 11.0 software (Systat Software, San Jose, CA, USA) and data were presented as mean ± standard derivation (SD) or as a percentage. For the analysis of the significance of differences, we used one-way analysis of variance (ANOVA) or two-tailed Student’s *t*-test. *P* values equal to or less than 0.05 were considered statistically significant.

## Results

### 1. Expression of GmCSF *in vitro*

**Fig 1:**
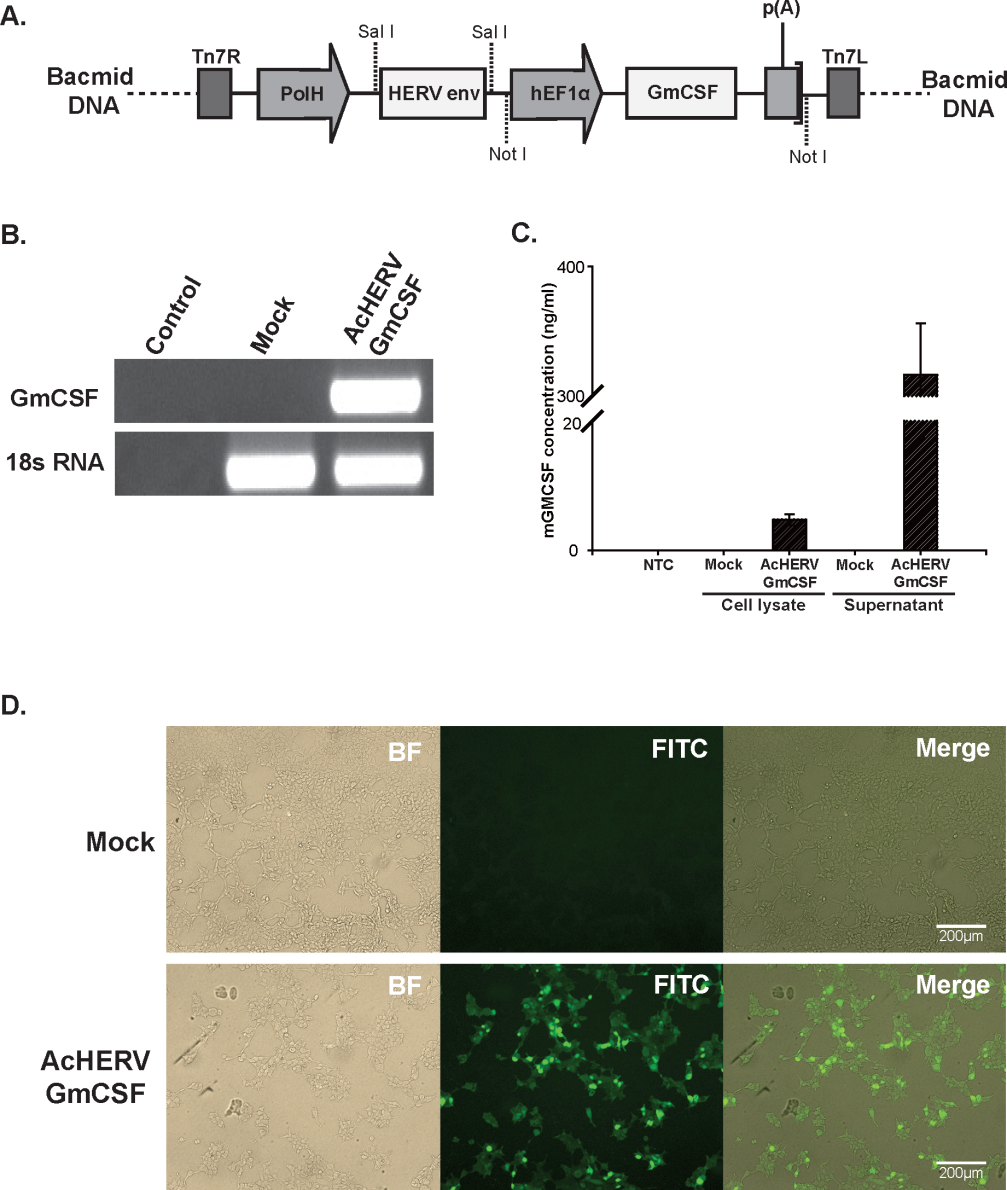
Diagram of the recombinant baculovirus, AcHERV-GmCSF, and its expression in mammalian cells. (A) Diagram of Bacmid DNA of AcHERV-GmCSF containing HERV envelope and GmCSF genes under the transcriptional control of the AcMNPV PolH and hEF1α promoters, respectively. (B) Detection of mRNAs for GmCSF and 18s rRNA in baculovirus-infected 293TT cells by RT-PCR. Lane 1: Control for RT-PCR; lane 2: mock infection with AcMNPV in 293TT cells; lane 3: AcHERV-GmCSF-infected 293TT cells. (C) Quantification of GmCSF expression in baculovirus-infected 293TT cell lysates and supernatants by ELISA. NTC, not treated control; Mock, AcMNPV baculovirus-infected cells; AcHERV-GmCSF, AcHERV-GmCSF-infected cells. (D) Fluorescence micrograph of baculovirus-infected 293TT cells. Seventy-two hours after infection, the cells were incubated with a monoclonal mouse antibody against GmCSF followed by incubation with a FITC-conjugated goat anti-mouse IgG antibody. Mock, AcMNPV-infected cells; AcHERV-GmCSF, AcHERV-GmCSF-infected cells; Merge, merged image.

To efficiently express GmCSF from baculovirus in mammalian cells, HERV coated baculovirus expressing GmCSF was constructed in Bacmid DNA containing HERV *env* and GmCSF under the control of the 5’-AcMNPV polyhedron promoter (PolH) and hEF1α promoter, respectively (Fig 1A). HERV glycoprotein coated baculovirus expressing GmCSF (AcHERV-GmCSF), produced in Sf9 cells, was capable of infecting 293TT cells. GmCSF mRNA (Fig 1B) and protein (Fig 1C) expression were detected in virus-infected 293TT cells by RT-PCR and ELISA, respectively. GmCSF protein was detected in both supernatants and cell lysates, but the majority was detected in supernatants. GmCSF protein expression in AcHERV-GmCSF-infected 293TT cells was further confirmed by immunofluorescence staining using a mouse monoclonal antibody against GmCSF and a FITC-conjugated anti-mouse antibody (Fig 1D). Collectively, these results demonstrate that GmCSF was successfully expressed in 293TT cells infected with AcHERV-GmCSF.

### 2. Changes in hematological composition induced by GmCSF

**Fig 2:**
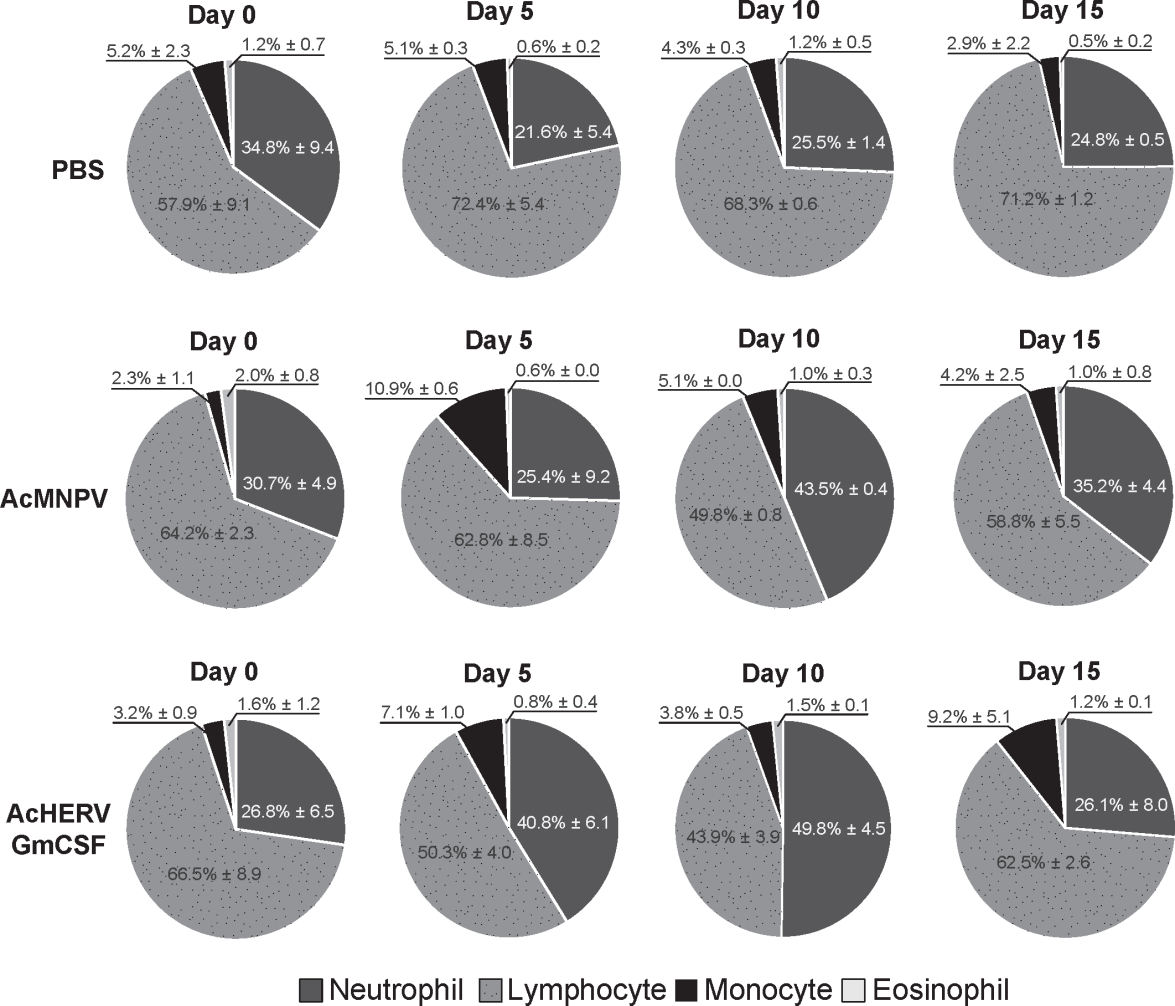
Hematological analysis of changes in white blood cell composition. Two samples (2/2) of blood from mice immunized with AcMNPV or AcHERV-GmCSF, or control mice injected with PBS, were collected from the jugular vein at 5-day intervals, and hematological analyses were performed. Neutrophils, dark gray; lymphocytes, dotted gray; monocytes, black; eosinophils, gray. Data were presented as mean percentage of leukocyte ± SD and pie graphs are presented mean percentage of leukocyte.

Hematological changes in BALB/c mice (n = 2/group, i.m.) injected with AcHERV-GmCSF were analyzed as described in Materials and Methods. Compared with PBS injection, immunization with wild-type baculovirus (AcMNPV) alone increased the percentage of monocytes from 2.3% ± 1.1 to 10.9% ± 0.6 on day 5, and increased the percentage of neutrophils to 43.5% ± 0.4 on day 10 (Fig 2). In mice immunized with AcHERV-GmCSF, however, neutrophils were increased to 40.8% ± 6.1 on day 5, a level similar to that achieved on day 10 in the AcMNPV-immunized group. Neutrophil levels in AcHERV-GmCSF immunized mice further increased to 49.8% ± 4.5 on day 10 (Fig 2), a level 2-fold higher than that in the PBS control group. These results indicate that expression of GmCSF together with the HERV coated baculovirus system has trend to increase in neutrophil levels that is apparent as early as 5 days post immunization.

### 3. Humoral immune response in mice

**Fig 3:**
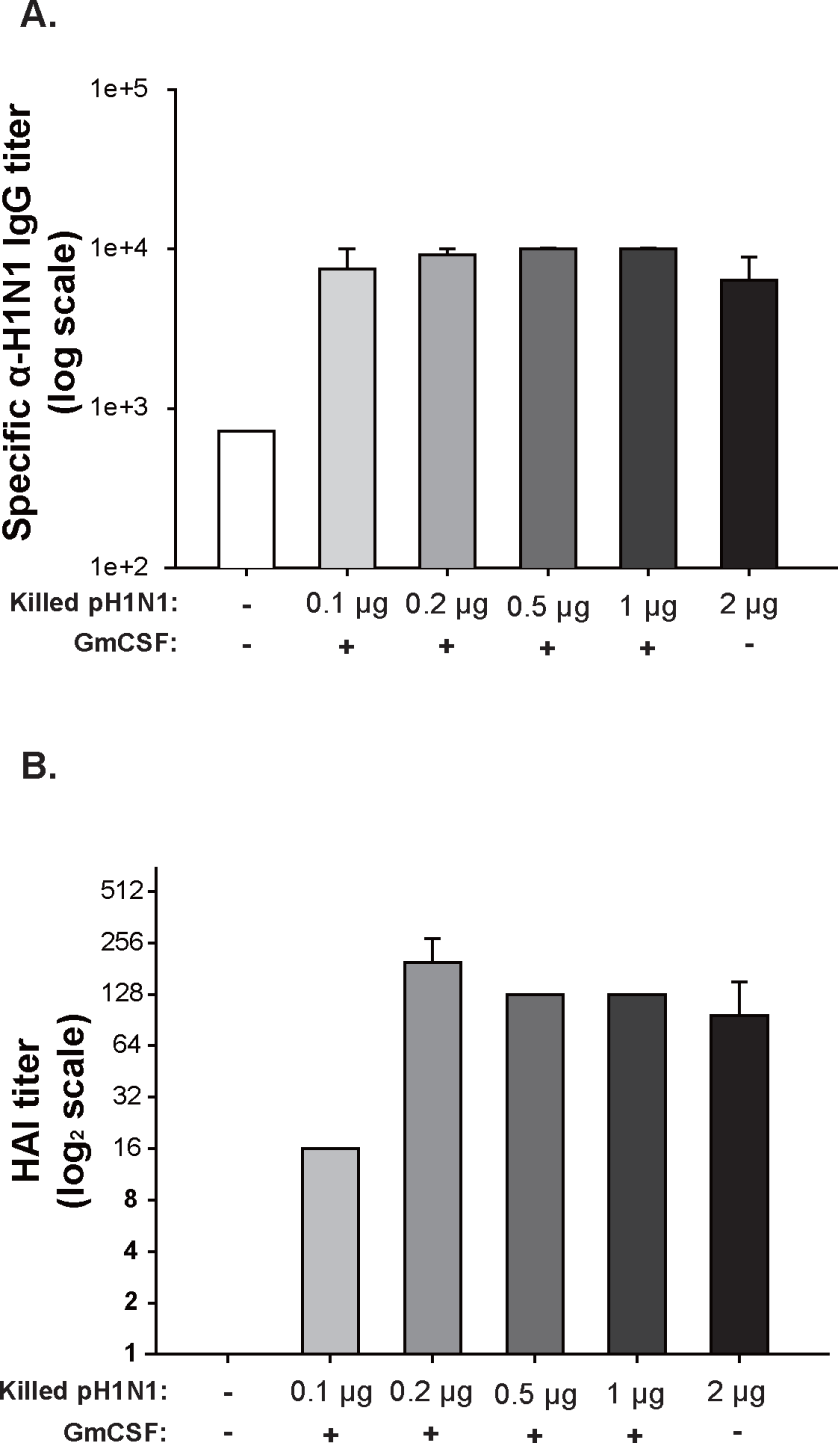
Determination of an effective dose of virus for killed vaccine. A dose of killed vaccine that was effective when used in conjunction with GmCSF was determined by intramuscularly injecting BALB/c mice with 2 μg of killed vaccine (positive control), serially diluted (1–0.1 μg) killed vaccine together with AcHERV-GmCSF (1×10^7^ FFU), or AcHERV-GmCSF (1×10^7^ FFU) alone. (A) Antigen-specific IgG antibody titers against pH1N1 (8 HAU) in mouse sera were determined by ELISA (3/3). (B) HAI response in mouse sera (3/3). Statistical analysis showed that data were not significant with *p*>0.05 (one way ANOVA).

Although 2 μg of killed vaccine has been reported to elicit a sufficient immune response and afford protection [21], an adjuvant is expected reduce the quantity of antigen needed for effective immunization. Thus, in preliminary experiments to evaluate the efficacy of AcHERV-GmCSF, we determined the minimum necessary dose of killed vaccine for use in conjunction with AcHERV-GmCSF. To this end, mice (n = 3/group, i.m.) were immunized with 2 μg of killed vaccine alone or with serially diluted (1–0.1 μg) killed vaccine together with AcHERV-GmCSF; as an additional control, mice were injected with AcHERV-GmCSF only. Two weeks after immunization, mouse sera were collected and the titer of pH1N1-specific IgG and mean hemagglutination (HAI) inhibition titers were analyzed by ELISA and HAI assay, respectively. As shown in Fig 3, mice immunized with 0.2 to 1 μg killed vaccine together with AcHERV-GmCSF had a comparable HAI titers, whereas mice immunized with 0.1 to 1 μg had similar HA-specific IgG titers. On the basis of these results, we selected 0.2 μg of killed vaccine, which when combined with AcHERV-GmCSF elicited an immune response similar to that previously reported for 2 μg alone, as the immunizing dose for subsequent experiments on the adjuvant properties of AcHERV-GmCSF.

**Fig 4:**
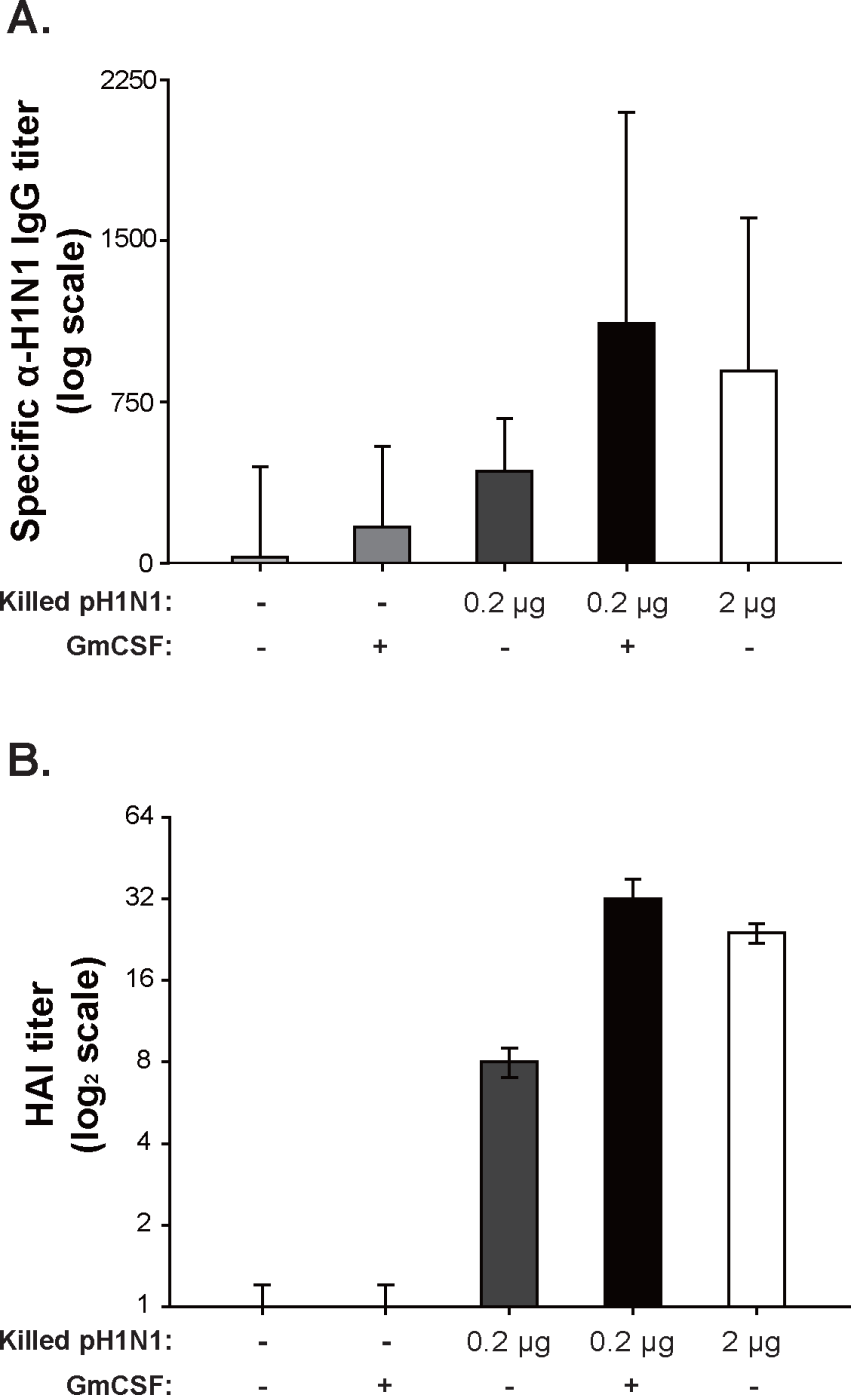
Humoral immune responses in mice immunized with killed vaccine alone and together with AcHERV-GmCSF. Sera from mice injected intramuscularly with PBS, AcHERV-GmCSF, killed vaccine alone, or killed vaccine together with AcHERV-GmCSF were collected 2 weeks after immunization and evaluated for humoral immune response. (A) Antigen-specific IgG antibody titers against pH1N1 (8 HAU) in mouse sera were determined by ELISA. (B) HAI titer in mouse sera. ELISA and HAI assays were performed using eight randomly selected samples from each group (8/16). Statistical analysis showed that data were significant with *p*<0.05 (one way ANOVA).

To investigate the adjuvant effect of AcHERV-GmCSF on immunogenicity elicited by killed vaccine in mice, we compared the humoral immune response in mice (n = 16/group, i.m.) immunized with 0.2 μg killed vaccine together with AcHERV-GmCSF (1×10^7^ FFU) with that in mice injected with 0.2 or 2 μg of killed vaccine alone using ELISA and HAI assays. Mice injected with PBS (100 μl) or AcHERV-GmCSF alone (1×10^7^ FFU) served as additional controls. As shown in Fig 4A, production of pH1N1-specific IgG mice was 1.5-fold higher in mice immunized with 0.2 μg killed vaccine together with AcHERV-GmCSF than in mice immunized with 0.2 μg killed vaccine alone. Similar results were observed for HAI titers, which were ~4-fold higher in immunized mice co-injected with AcHERV-GmCSF than in mice immunized with 0.2 μg of killed vaccine only (Fig 4B). pH1N1-specific IgG and HAI titers in the group co-injected with 0.2 μg killed vaccine and AcHERV-GmCSF were even higher than in those injected with 2 μg of killed vaccine—the currently established dose. Collectively, these data clearly demonstrate that co-injection of GmCSF efficiently boosts the production of antibodies against pH1N1.

### 4. Cellular immune response in mice

**Fig 5:**
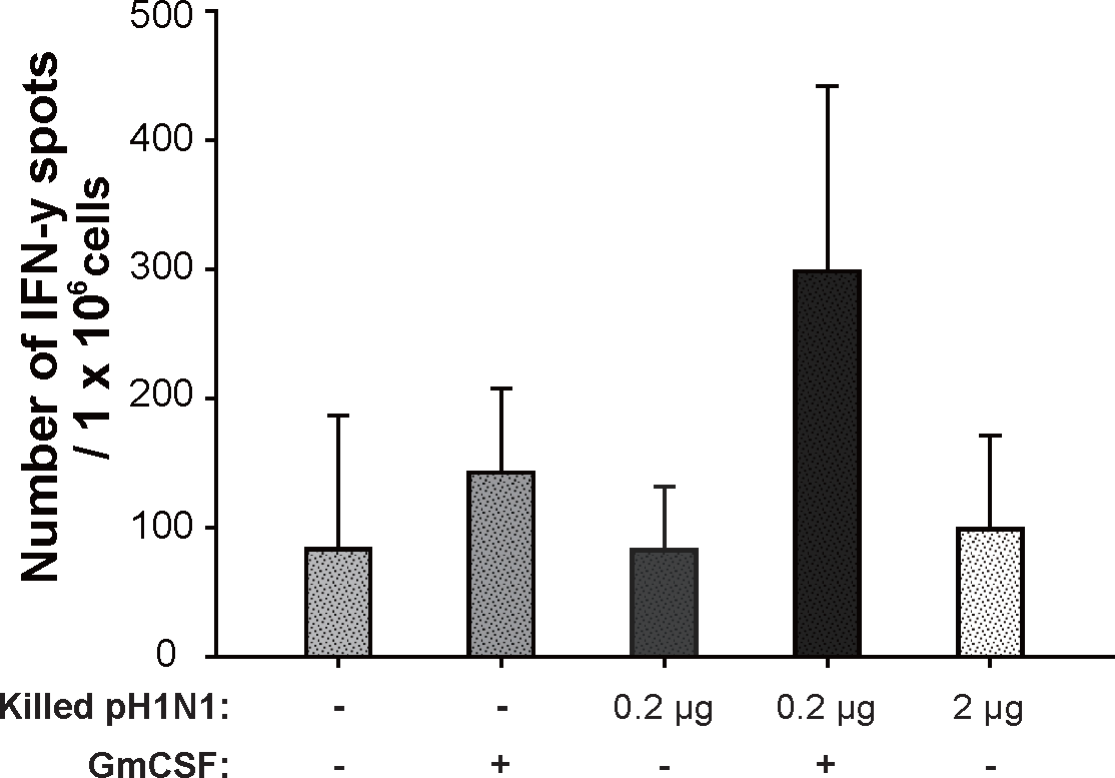
Analysis of IFN-γ production in mice immunized with killed virus vaccine together with AcHERV-GmCSF. The number of IFN-γ spots from pH1N1-specific T cells in splenocytes (3/16) 2 weeks post immunization were analyzed using an ELISPOT assay. Statistical analysis showed that data were significant with *p*<0.05 (one way ANOVA).

To determine the effect of GmCSF on IFN-γ production, we performed ELISPOT assays on splenocytes isolated from mice (3/16). Splenocytes from mice immunized with 2 μg of killed vaccine produced only basal levels of IFN-γ, similar to results obtained in mice injected with PBS (Fig 5). However, splenocytes from mice immunized with 0.2 μg of killed vaccine together with AcHERV-GmCSF produced 3-fold more IFN-γ than those from mice immunized with 2 μg of killed vaccine alone. These results support the conclusion that GmCSF is a strong candidate adjuvant with the potential to increase the cellular immune response in addition to the humoral immune response.

### 5. Protection against pH1N1 viral challenge in mice

**Fig 6:**
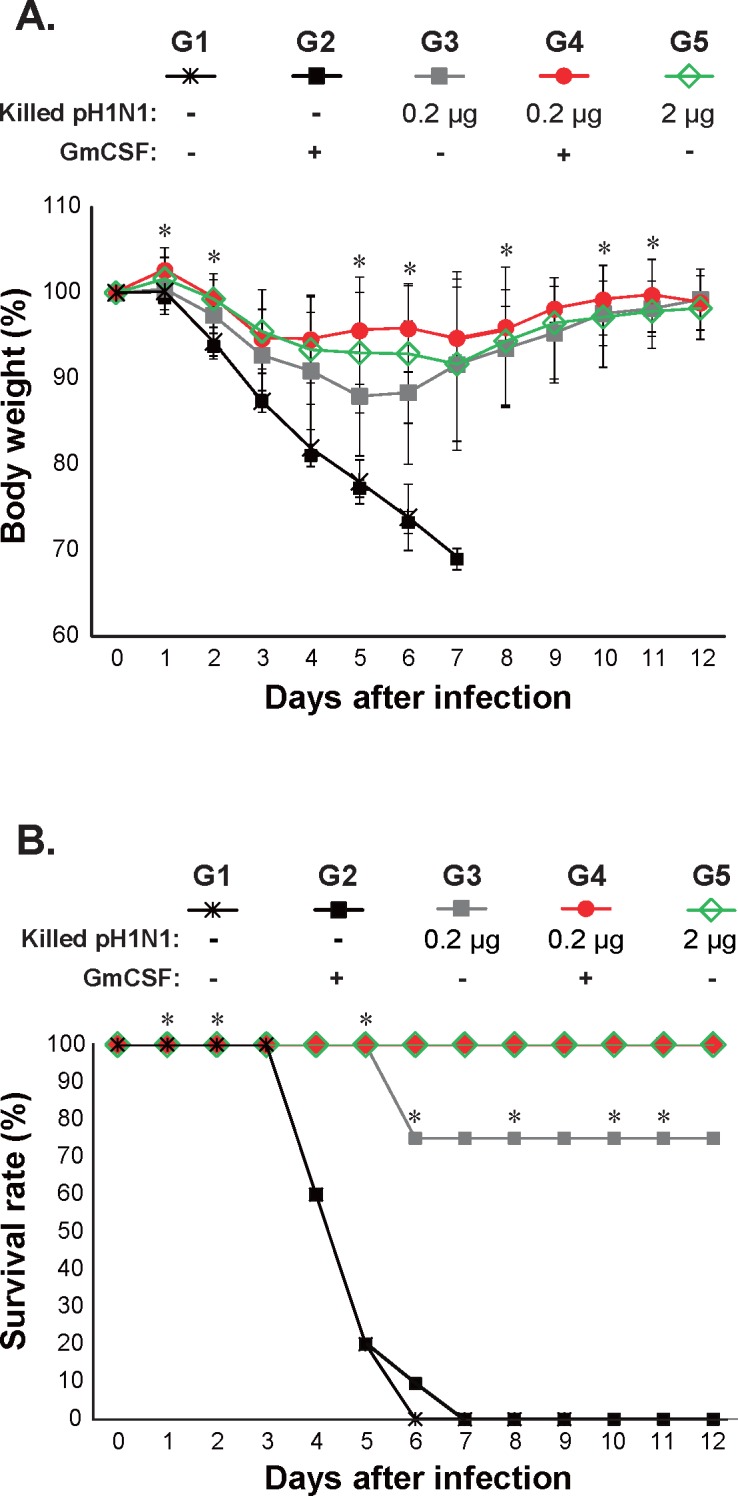
Protective effect of immunization against challenge with a lethal dose of ma-pH1N1. The body weight of mice intranasally challenged with a 10LD_50_ dose of mouse-adapted influenza virus (ma-pH1N1) 4 weeks after the final immunization was monitored for 12 consecutive days. (A) Percentage body weight change after challenge with a 10LD_50_ dose of mouse-adapted influenza virus (ma-pH1N1). Changes in body weight (n = 9 mice/group) are expressed as the mean ± SD for each group. (B) Survival rate after challenge with a 10LD_50_ dose of mouse-adapted influenza virus (ma-pH1N1). Crosses, mice injected with PBS; black squares, mice vaccinated with AcHERV-GmCSF; gray squares, mice vaccinated with 0.2 μg of killed vaccine; red circles, mice vaccinated with 0.2 μg of killed vaccine together with AcHERV-GmCSF; open green diamonds, mice vaccinated with 2 μg of killed vaccine. Statistical analysis performed between the +GmCSF (0.2 μg of killed vaccine) and the–GmCSF (0.2 μg of killed vaccine). Statistical analysis showed that data were significant with *p < 0.05 (two-tailed Student’s *t*-test).

Mice (n = 13/group) were intranasally challenged with a 10LD_50_ dose of infectious pH1N1 virus 3 weeks after immunization, one day after virus challenge, 4 mice from each group were separated for lung titer measurement and histological analysis. The remaining 9 mice per group were monitored daily for weight loss. Control mice injected with PBS or AcHERV-GmCSF alone exhibited a weight loss of approximately 25% upon euthanizing (6 and 7 days post challenge). In contrast, mice immunized with killed vaccine alone (2.0 μg) or killed vaccine (0.2 μg) together with AcHERV-GmCSF, showed no significant weight loss (Fig 6A). A 12-day monitoring period showed that the absence of body weight loss in immunized mice after viral challenge was correlated with survival. Mice in groups injected with 2 μg of killed vaccine or co-injected with 0.2 μg killed vaccine together with AcHERV-GmCSF, both of which maintained their body weight, exhibited a survival rate of 100%. Even among mice in the group injected with 0.2 μg of killed vaccine only—a group that showed approximately a 12% loss of body weight by day 5–6—a majority survived (Fig 6). In contrast, control mice injected with PBS or AcHERV-GmCSF only exhibited severe illness, characterized by huddling, ruffled fur, lethargy and anorexia, leading to humane endpoint (e.g. weight loss up to 25% of their initial weight) on days 6 and 7 (Fig 6).

### 6. Histological analysis of lungs from immunized mice after viral challenge

One day after virus challenge, 4 mice from each group were separated for lung titer measurement and histological analysis. To assess the relationship between the titer of collected virus and histological lesions in the lung from separated mice, mice were sacrificed and the lung samples in PBS group and in the rest of groups were collected on day 6 and 7, respectively. The reason that the lung samples in PBS group were collected on day 6, mice showed severe moribund condition and also reached humane endpoint of weight loss, and then determined virus titer and lung damage. Virus titer was determined by quantifying infection of MDCK cells, expressed as log_10_ TCID_50_/ml (Table 1).

**Table 1:** Viral recovery in lung tissue of mice challenged with pH1N1.

Immunization group^a^	Lung virus titers^b^log_10_ TCID_50_/ml tissue
**PBS**	7.8 ± 0.63(4/4)
**AcHERV-GmCSF**	9.0 ± 0.65(4/4)
**0.2 μg of killed vaccine**	5.8 ± 0.61(2/4)
**0.2 μg of killed vaccine + AcHERV-GmCSF**	<3.1(0/4)
**2 μg of killed vaccine**	5.5 ± 0.63(2/4)

^a^Groups of 13 mice were intramuscularly immunized as described in Section 2.6.

^b^Mice were intranasally challenged with a 10x LD_50_ dose of pH1N1 on 7 days post immunization. Mice were sacrificed and the lung samples in PBS group and in the rest of groups were collectedexamined on day 6 and 7, respectively.

Viral titers in lung homogenates were determined as described in Section 2.8.

Virus titers were determined by infection of MDCK cells, and are expressed as log_10_ TCID_50_/ml. Data were presented as means ± SD of titers of samples. The number of mice that shed virus is indicated in parentheses (number of mice shedding virus/number of mice tested).

**Fig 7:**
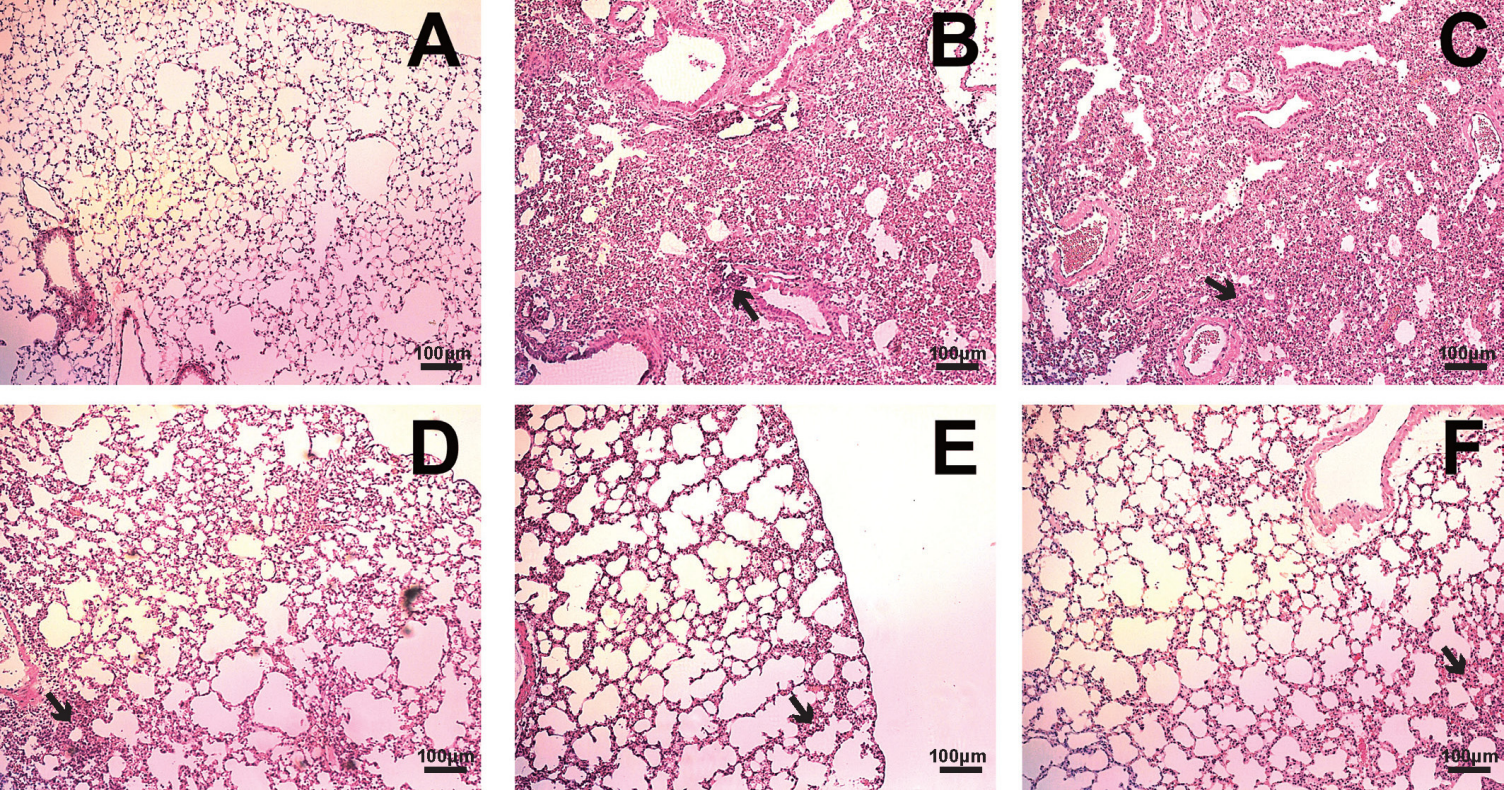
Histological lesions in lung sections from immunized mice after challenge with pH1N1. Separated mice (n = 4 mice/group) from each group were sacrificed 6 or 7 days post challenge, and their lungs were HE-stained for histological evaluation. (A) Non-infected BABL/c mice; (B) mice injected with PBS; (C) mice injected with AcHERV-GmCSF; (D) mice vaccinated with 0.2 μg of killed vaccine; (E) mice vaccinated with 0.2 μg of killed vaccine together with AcHERV-GmCSF; and (F) mice vaccinated with 2 μg of killed vaccine. Arrows indicated the infiltration of inflammatory cells, including the infiltration in the vessels, in the pulmonary parenchyma, and in the alveolar septa. Scale bar, 100 μm.

The amount of virus from the PBS-injected mice was 10^7.8 ± 0.63^, whereas that in mice immunized with 0.2 or 2 μg killed vaccine was 10^5.8 ± 0.61^ and 10^5.5± 0.63^, respectively—an overall 2log10 reduction in viral titer. Strikingly, viruses in mice in the group immunized with 0.2 μg killed vaccine and co-injected with AcHERV-GmCSF were reduced to an undetectable level. Consistent with viral titer results, histological analyses of hematoxylin & eosin-stained lung sections revealed that mice injected with PBS or AcHERV-GmCSF alone had severe infiltration in the vessels, bronchioles and alveoli; even mice immunized with 0.2 μg killed vaccine alone showed interstitial/alveolar infiltration and structural damage around bronchioles or vessels (Fig 7B–7D). However, mice immunized with 0.2 μg killed vaccine together with AcHERV-GmCSF or immunized with 2 μg of killed vaccine alone had less interstitial and alveolar infiltration, suggesting protection of these structures against viral infection (Fig 7E and 7F).

## Discussion

The development of adjuvants is considered as important as the determination antigens or epitopes that are effective in promoting activation of immune responses and production of appropriate cytokines [28]. Recent trends suggest inclusion of cytokines in the modern classification of adjuvants. The use of cytokines as vaccine adjuvants is appealing because they may act as chemoattractants for immune cells and may further augment the vaccine's protective effects; their effects may also allow one to choose which arm of the immune response is enhanced [29].

Hematopoietic cytokines have been demonstrated to stimulate formation of neutrophil, monocyte-macrophage and eosinophil colonies [30–32], and enhance primary immune responses by activating and recruiting antigen presenting cells (APC) and antibody (Ab)-dependent cell-mediated cytotoxicity [10]. Thus, it has been postulated that GmCSF, as a hematopoietic cytokine, would be an effective adjuvant in preventing influenza viral infection [33].

Previous research has demonstrated that GmCSF promotes proliferation of hematopoietic cells, especially neutrophils, of the inflammatory signaling network [31, 32], prompting us to investigate the effects of GmCSF on hematopoietic composition and neutrophil levels. It has been shown that baculovirus infection in and of itself is immunogenic, affecting the proliferation of monocytes/macrophages, which act through nitric oxide (NO) production to play an important role in the innate immune response responsible for antiviral and bactericidal activity [34, 35].

In addition to the hematological changes induced by wild type baculovirus infection alone. However, on days 5, AcHERV-GmCSF has shown faster neutrophil recruitment than AcMNPV (40.8 ± 6.1 vs 25.4 ± 9.2) and further increased neutrophil proportion up to 50% in the blood by 10 days post immunization, providing strong evidence that AcHERV-GmCSF functions as an efficient recruiter of neutrophils and rapid promoter of subsequent changes in hematological composition. The hematological changes induced by immunization with AcHERV-GmCSF sets the stage for the trend of AcHERV-GmCSF as an efficient adjuvant. In addition, the increased humoral responses and IFN-γ secretion in mice immunized with killed vaccine and co-injected with AcHERV-GmCSF (Figs 4 and 5) indicate that AcHERV-GmCSF induces B-cell responses that produce antibodies specific to influenza pH1N1, stimulates IFN-γ secretion in splenocytes, and enhances the level of T cell responses.

More evidence for GmCSF as an adjuvant is provided by the fact that virally challenged mice maintained their body weight, suggesting enhanced resistance to influenza virus and an efficient immune response (Fig 6). Other signs of rapid recovery from infection include the low viral titer and low viral shedding in the lung, as determined subsequently based on TCID_50_ and pneumonectomy [36]. These findings provide support for the conclusion that immunization with influenza vaccine together with AcHERV-GmCSF protects against pandemic influenza virus by enhancing the primary immune response and promoting a high level of IFN-γ secretion.

These results provide strong evidence that AcHERV-GmCSF can be used as a vaccine adjuvant for various purposes, such as enhancing the immunogenicity of highly purified or recombinant antigens and reducing the amount of antigen or number of immunizations needed to achieve protective immunity. Additionally, the baculoviral vector containing GmCSF has several advantages, including ease of manipulation, simple scale-up, and lack of toxicity.

Taken together, our findings indicate that the baculovirus-based AcHERV-GmCSF vaccine adjuvant together with a small quantity (0.2 μg) of killed vaccine exert a strong protective effect that results from sufficient neutrophil recruitment, IFN-γ secretion, IgG production, and low viral shedding. The protective efficacy is comparable to that observed in mice immunized with a 10-times higher dose (2 μg) of killed virus vaccine alone.

Thus, the strong humoral and cellular immune responses shown in this study suggest that (1) a HERV envelope-coated, recombinant baculoviral vector encoding cytokines as a vaccine adjuvant is an efficient vector system for reducing the dose of killed vaccine required to about 1/10 the currently used dose; and (2) GmCSF has considerable advantages for applications as a new vaccine adjuvant with the potential to reduce morbidity and mortality associated with new influenza virus epidemics.

## Supporting Information

S1 ARRIVE Checklist NC3Rs ARRIVE Guidelines checklist. (DOCX)

S1 Fig Experimental timelines. (A) BALB/c mice were given 1×10^7^ focus-forming units (FFU) of AcHERV-GmCSF or AcMNPV (1×10^7^ FFU) or PBS (100 μl) (↑). Four samples of blood were collected at 5-day intervals from the jugular vein of individual mice into tubes containing K2 EDTA (↓). (B) BALB/c mice were immunized by intramuscular injection of serially diluted (1.0–0.1 μg), killed vaccine together with 1×10^7^ FFU AcHERV-GmCSF; as a control, mice were immunized with 2 μg of killed vaccine or 1×10^7^ FFU AcHERV-GmCSF only at the same time points (↑). Blood was collected from the jugular vein of individual mice into 1.6 ml tube (↓). (C) BALB/c mice were divided into five immunization groups: (1) PBS control (100 μl), (2) AcHERV-GmCSF only (1×10^7^ FFU), (3) low-dose vaccine only (0.2 μg killed vaccine), (4) high-dose vaccine only (2.0 μg killed vaccine), and (5) vaccine plus AcHERV-GmCSF adjuvant (0.2 μg killed vaccine together with 1×10^7^ FFU AcHERV-GmCSF) and given i.m. injection (↑). On days 7, 14, 20 or 21 blood collection, splenectomy and pnemonectomy were proceeded, respectively (↓). Two weeks after immunization, mice were transferred to a biological safety level 2 facility, where they were sedated and challenged intranasally with mouse-adapted influenza virus A/CA/04/2009 (ma-pH1N1) at a 10×LD_50_ dose (▲). Mice were observed health condition and weighed for 12 consecutive days.
(TIFF)
