# Supplementary material for: Effect of AcHERV-GmCSF as an Influenza Virus Vaccine Adjuvant
Source: PLoS One. 2015 Jun 19;10(6):e0129761. doi: 10.1371/journal.pone.0129761 (PMC4475044; doi:10.1371/journal.pone.0129761)

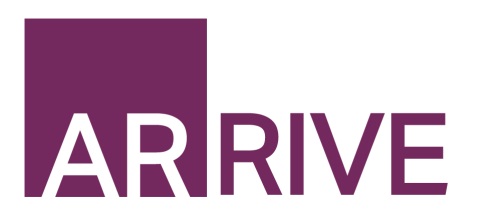


The ARRIVE Guidelines Checklist

Animal Research: Reporting In Vivo Experiments

Carol Kilkenny^1^, William J Browne^2^, Innes C Cuthill^3^, Michael Emerson^4^ and Douglas G Altman^5^

*^1^The National Centre for the Replacement, Refinement and Reduction of Animals in Research, London, UK, ^2^School of Veterinary Science, University of Bristol, Bristol, UK, ^3^School of Biological Sciences, University of Bristol, Bristol, UK, ^4^National Heart and Lung Institute, Imperial College London, UK, ^5^Centre for Statistics in Medicine, University of Oxford, Oxford, UK.*

|  | ITEM | RECOMMENDATION | Section/ Paragraph |
| --- | --- | --- | --- |
| 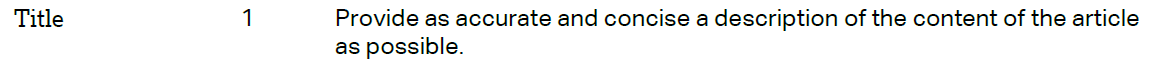 | | | **Title** |
| 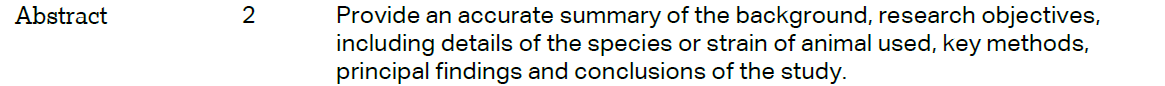 | | | **Abstract/**  **Paragraph 1**  **Paragraph 2**  **Paragraph 3** |
| INTRODUCTION | | |  |
| 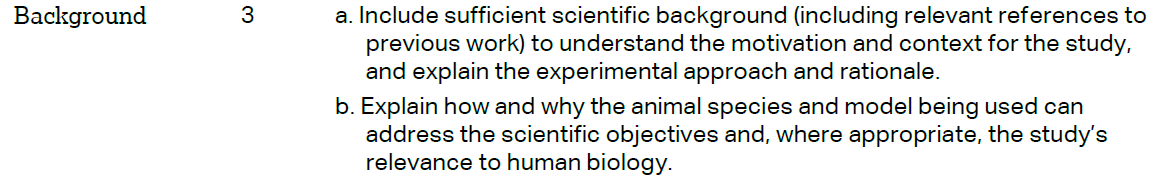 | | | **Introduction/**  **Paragraph 1**  **Paragraph 2**  **Paragraph 3**  **Paragraph 4**  **Paragraph 5** |
| 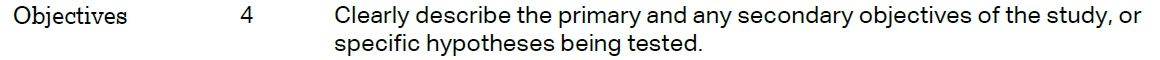 | | | **Introduction/**  **Paragraph 5** |
| METHODS | | |  |
| 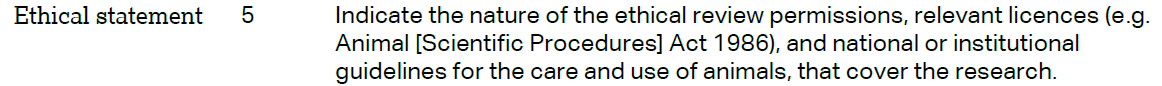 | | | **Methods/**  **Sub-section 1** |
| 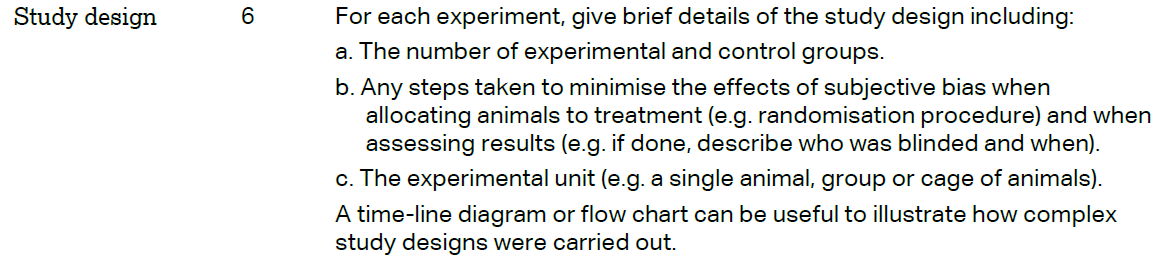 | | | **Methods/**  **Sub-section 3**  **Sub-section 6** |
| 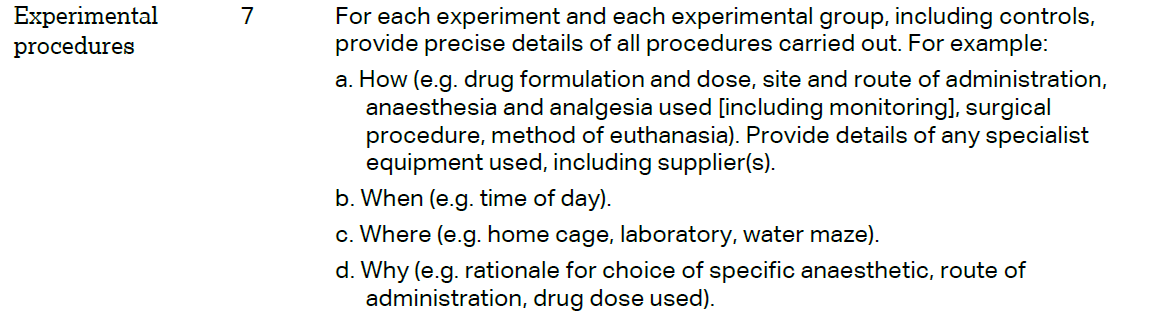 | | | **Methods/**  **Sub-section 3**  **Sub-section 6** |
| 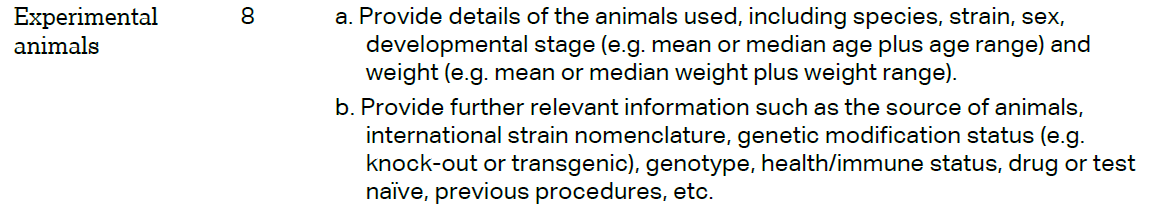 | | | **Methods/**  **Sub-section 3** |

The ARRIVE guidelines. Originally published in *PLoS Biology*, June 2010^1^

| 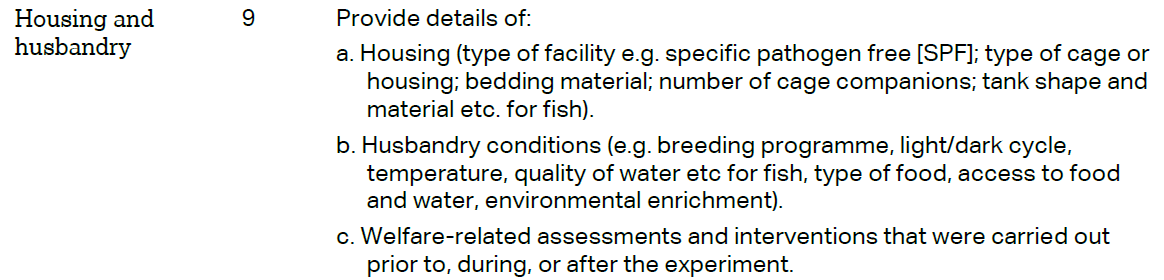 | **Methods/**  **Sub-section 3** |  |
| --- | --- | --- |
| 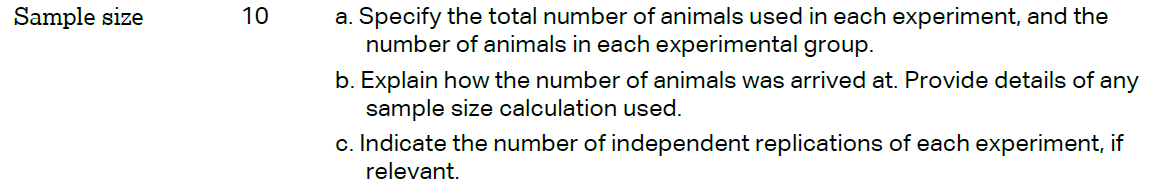 | **Methods/**  **Sub-section 6** |  |
| 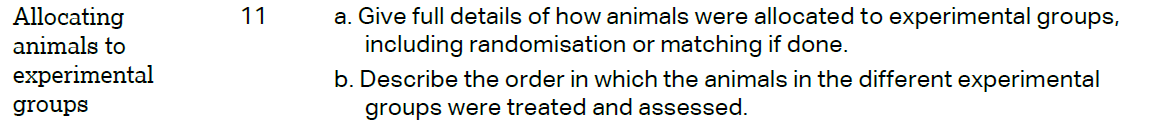 | **Methods/**  **Sub-section 6**  **Sub-section 8** |  |
| 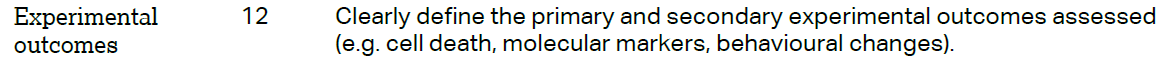 | **Methods/**  **Sub-section 6**  **Sub-section 7**  **Sub-section 8** |  |
| 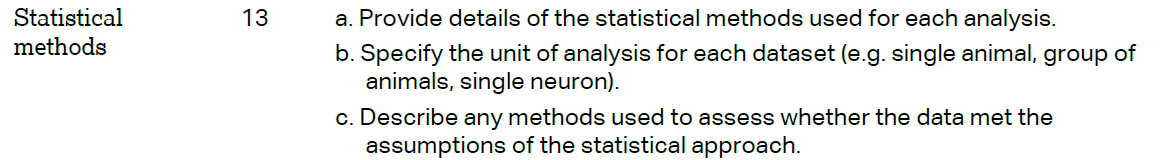 | **Methods/**  **Sub-section 9** |  |
| RESULTS |  |  |
| 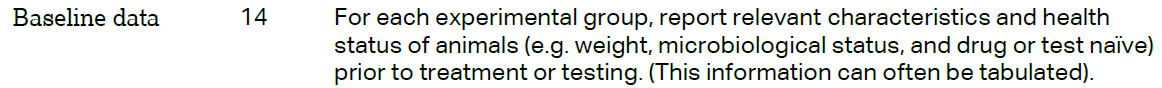 | **Methods/**  **Sub-section 3** |  |
| 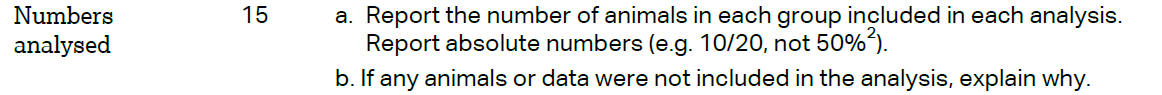 | **Results/**  **Sub-section 2**  **Sub-section 3**  **Sub-section 4**  **Sub-section 5**  **Sub-section 6** |  |
| 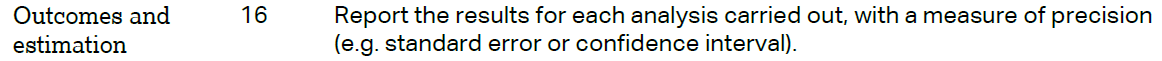 | **Results/**  **Sub-section 2**  **Sub-section 3**  **Sub-section 4**  **Sub-section 5**  **Sub-section 6** |  |
| 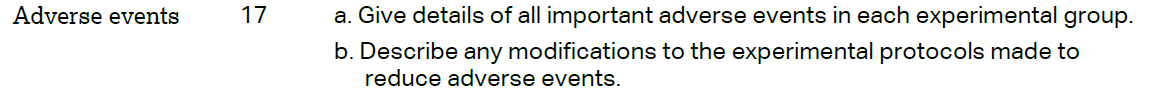 | **Results/**  **Sub-section 6** |  |
| DISCUSSION |  |  |
| 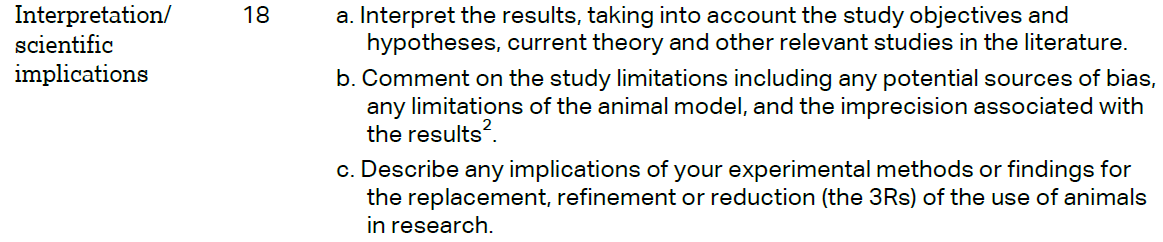 | **Discussion/**  **Paragraph 4**  **Paragraph 5**  **Paragraph 6** |  |
| 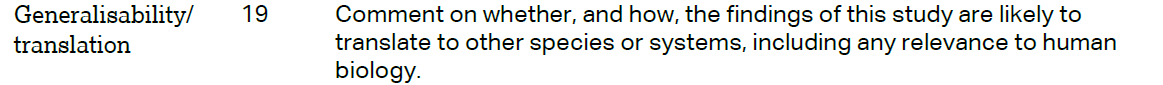 | **Discussion/**  **Paragraph 6**  **Paragraph 7**  **Paragraph 8** |  |
| 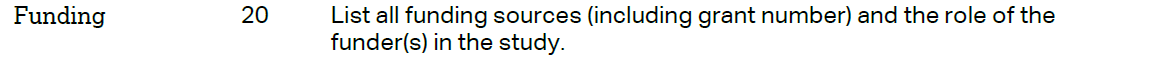 | **Provide this information during the manuscript submission process** | |


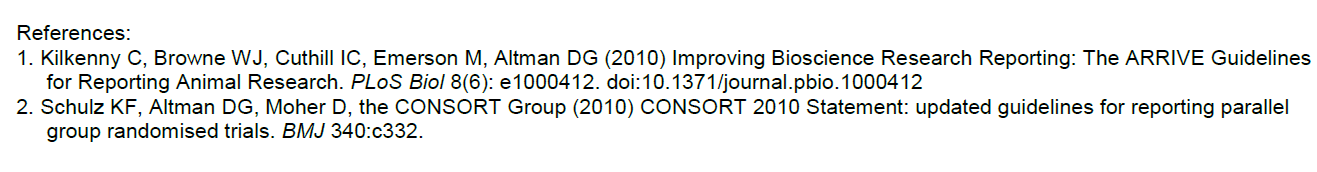

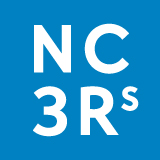

Supplement: S1 ARRIVE Checklist — (DOCX) [file pone.0129761.s001.docx]
