# Supplementary figures and images for: Effect of AcHERV-GmCSF as an Influenza Virus Vaccine Adjuvant
Source: PLoS One. 2015 Jun 19;10(6):e0129761. doi: 10.1371/journal.pone.0129761 (PMC4475044; doi:10.1371/journal.pone.0129761)

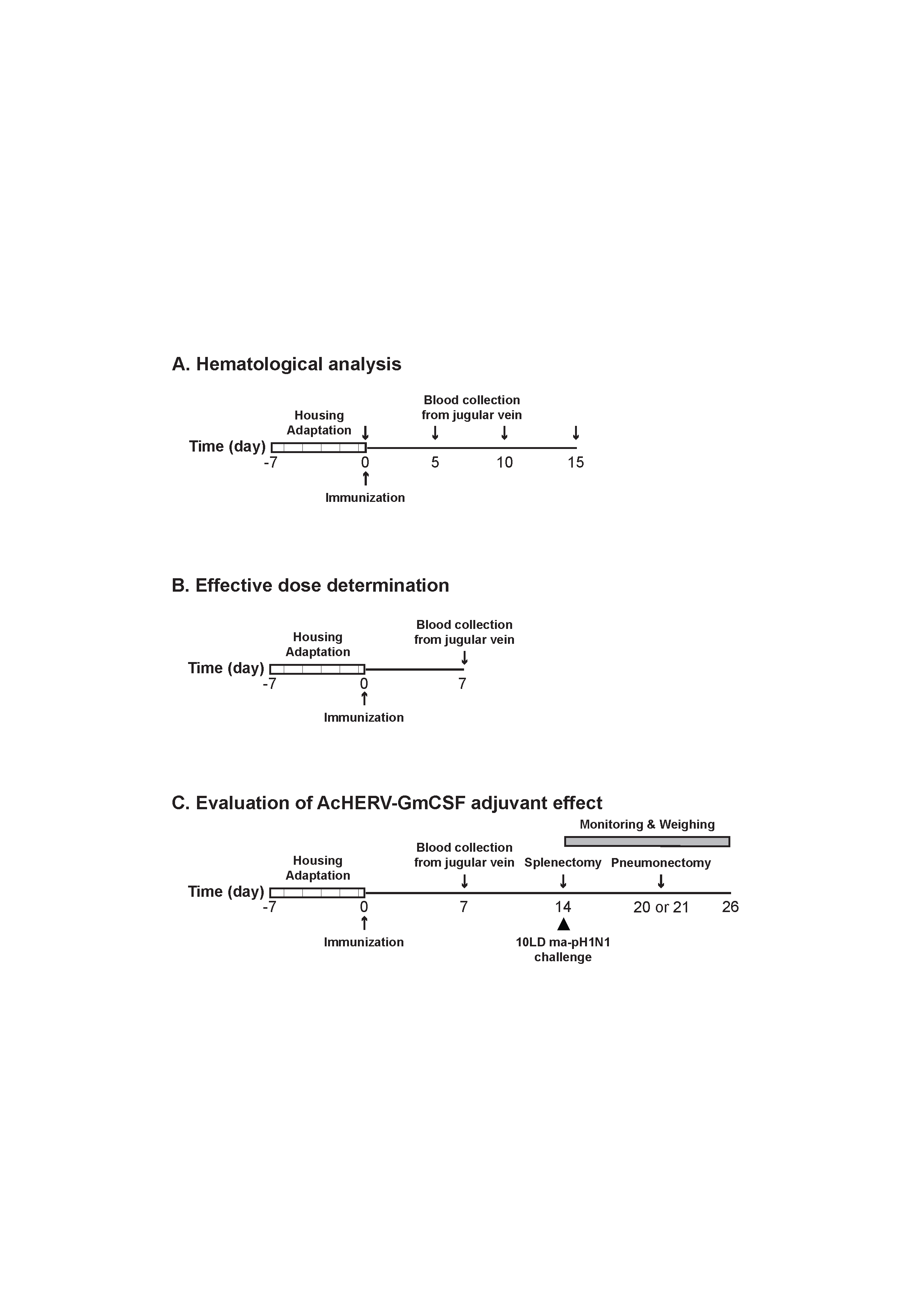

Supplement: S1 Fig — (A) BALB/c mice were given 1×10^7^ focus-forming units (FFU) of AcHERV-GmCSF or AcMNPV (1×10^7^ FFU) or PBS (100 μl) (↑). Four samples of blood were collected at 5-day intervals from the jugular vein of individual mice into tubes containing K2 EDTA (↓). (B) BALB/c mice were immunized by intramuscular injection of serially diluted (1.0–0.1 μg), killed vaccine together with 1×10^7^ FFU AcHERV-GmCSF; as a control, mice were immunized with 2 μg of killed vaccine or 1×10^7^ FFU AcHERV-GmCSF only at the same time points (↑). Blood was collected from the jugular vein of individual mice into 1.6 ml tube (↓). (C) BALB/c mice were divided into five immunization groups: (1) PBS control (100 μl), (2) AcHERV-GmCSF only (1×10^7^ FFU), (3) low-dose vaccine only (0.2 μg killed vaccine), (4) high-dose vaccine only (2.0 μg killed vaccine), and (5) vaccine plus AcHERV-GmCSF adjuvant (0.2 μg killed vaccine together with 1×10^7^ FFU AcHERV-GmCSF) and given i.m. injection (↑). On days 7, 14, 20 or 21 blood collection, splenectomy and pnemonectomy were proceeded, respectively (↓). Two weeks after immunization, mice were transferred to a biological safety level 2 facility, where they were sedated and challenged intranasally with mouse-adapted influenza virus A/CA/04/2009 (ma-pH1N1) at a 10×LD50 dose (▲). Mice were observed health condition and weighed for 12 consecutive days. (TIFF) [file pone.0129761.s002.tiff]
